# Insecticide Activity of Essential Oils of *Mentha longifolia*, *Pulicaria gnaphalodes* and *Achillea wilhelmsii* Against Two Stored Product Pests, the Flour Beetle, *Tribolium castaneum*, and the Cowpea Weevil, *Callosobruchus maculatus*

**DOI:** 10.1673/031.012.7301

**Published:** 2012-07-01

**Authors:** Abbas Khani, Javad Asghari

**Affiliations:** ^1^ Department of Plant Protection, Faculty of Agriculture, University of Zabol, Zabol, Iran; ^2^ Jihad and Agriculture Organization of South Khorasan, Birjand, Iran

**Keywords:** fumigant toxicity, gas chromatography-mass spectrometry, mono terpenoids

## Abstract

Essential oils extracted from the foliage *of Mentha longifolia* (L.) (Lamiales: Lamiaceae) and *Pulicaria gnaphalodes* Ventenat (Asterales: Asteraceae), and flowers of *Achillea wilhelmsii* C. Koch (Asterales: Asteraceae) were tested in the laboratory for volatile toxicity against two storedproduct insects, the flour beetle, *Tribolium castaneum* Herbst (Coleoptera: Tenebrionidae) and the cowpea weevil, *Callosobruchus maculatus* F. (Coleoptera: Bruchidae). The chemical composition of the isolated oils was examined by gas chromatography-mass spectrometry. InM *longifolia,* the major compounds were piperitenon (43.9%), tripal (14.3%), oxathiane (9.3%), piperiton oxide (5.9%), and d-limonene (4.3%). In *P. gnaphalodes,* the major compounds were chrysanthenyl acetate (22.38%), 2L -4L-dihydroxy eicosane (18.5%), verbenol (16.59%), dehydroaromadendrene (12.54%), β-pinen (6.43%), and 1,8 cineol (5.6%). In *A. wilhelmsii,* the major compounds were 1,8 cineole (13.03%), caranol (8.26%), alpha pinene (6%), farnesyl acetate (6%), and p-cymene (6%). *C maculatus* was more susceptible to the tested plant products than *T castaneum.* The oils of the three plants displayed the same insecticidal activity against *C. maculatus* based on LC_50_ values (between 1.54µl/L air in *P. gnaphalodes,* and 2.65 µl/L air in *A. wilhelmsii*). While the oils of *A. wilhelmsii* and *M. longifolia* showed the same strong insecticidal activity against *T. castaneum* (LC_50_ = 10.02 and 13.05 µl/L air, respectively), the oil of *P. gnaphalodes* revealed poor activity against the insect (LC_50_ = 297.9 µl/L air). These results suggested that essential oils from the tested plants could be used as potential control agents for stored-product insects.

## Introduction

The global pest-harvest grain losses by insect damage and other bio-agents range from 10% to 40% (Papachristos and Stamopoulos 2002). Chemicals largely used as pesticides in crop protection could have undesirable effects such as ozone depletion, environmental pollution, toxicity to non-target organisms, pest resistance, pesticide residues, and direct toxicity to users (Isman 2006). With heightened concern for environmental problems and human health, the search for readily biodegradable and environmentally friendly insecticides is of interest among scientists (Shaaya et al. 1997; Isman 2000). Plants offer an alternative source of insectcontrol agents because they contain a range of bioactive chemicals, many of which are selective and have little or no harmful effect on non-target organisms and the environment (Shaaya et al. 1997; Rajendran and Sriranjini 2008).

The genus *Mentha* belongs to the family Lamiaceae (Labiatae), and consists of about 25-30 species, most of which are found in temperate regions of Eurasia, Australia and South Africa (Lange and Croteau 1999). *Mentha longifolia* (L.) (Lamiaceae), commonly known as wild mint, is a perennial herb that can grow 1–2 m high. Various biological activities have been reported for some species of *Mentha,* such as antibacterial (Oyedeji and Afolayan 2006; Hajlaoui et al. 2008), antifungal (Bouchra et al. 2003), and insecticidal properties (Franzios et al. 1997; Lamiri et al. 2001; Pavela 2005; Saljoqi et al. 2006). The oils of *M. longifolia* are known to contain numerous monoterpenoids with piperitone oxide, piperitone, piperitenone, pulegone, d-limonene, carvone, menthone, âcaryophyllene, 1,8-Cineole, and menthol as dominating compounds; however, there have been some variations in the constituents of this oil from different countries, and a chemogeographical variation has been observed in essential oil composition of this species (Oyedeji and Afolayan 2006).

The *Pulicaria* genus belongs to the family Compositae (Asteraceae), tribe Inuleae, which contains more than 77 species that are widely distributed throughout Asia, Europe and Africa (Anderberg 1991). The chemical investigation of the genus showed the presence of terpenes such as monoterpenes and oxygenated monoterpenes (Weyerstahl et al. 1999), diterpenes (Muhammad et al. 1992), and sesquiterpenes (Weyerstahl et al. 1999; Dendougui et al. 2000). Various biological activities have been reported for some species of *Pulicaria,* such as antibacterial, antifungal (El-Kamali et al. 1998; Bahman et al. 2002; Liu et al. 2010), and insecticidal properties (Ross et al. 1997; Dubaie and El-Khulaidi 2005).

The herb *Achillea,* which belongs to the family Compositae (Asteraceae), is a genus with more than 100 species around the world. These plants are medicinal perennial rhizomous herbs, native to Europe and Western Asia, but also found in Australia, New Zealand, and North America (Chevallier 1996). Previous research has investigated the chemical composition of the essential oil of *Achillea,* such as its antibacterial (Barel et al. 1991; Magiatis et al. 2002) and insecticidal properties (Calmasur et al. 2006; Jovanovic et al. 2007; Magdy and Samir 2008). Previous work showed that the essential oil extracted from *Achillea wilhelmsii* C. Koch (Asteraceae) leaves exhibited volatile toxicity to *Sitophilus granarius* and *Tribolium confusum* (Calmasur et al. 2006).

In the present study, the chemical components of essential oils from *M. longifolia* L. (Lamiaceae) and *P. gnaphalodes* vent. (Asteraceae) aerial parts, and *A. wilhelmsii* C. Koch (Asteraceae) flowers, were determined, and the insecticidal activity of them was tested against the adult stages of the stored-products pests, *Tribolium castaneum* Herbst (Coleoptera: Tenebrionidae) and *Callosobruchus maculatus* F. (Coleoptera: Bruchidae). No study has been reported concerning the activity of the three test oils as fumigants against these stored product insects.

## Materials and Methods

### Insect material

*C. maculatus* and *T. castaneum* were reared in plastic containers (20 cm length, 14 cm width, and 8 cm height, covered by a fine mesh cloth for ventilation) containing bean grain and wheat flour mixed with yeast (10:1, w/w), respectively. The culture was maintained in the dark, in a growth chamber set at 27±1°C and 65±5 relative humidity. All experiments were carried out under the same environmental conditions.

### Plants and essential oils

Aerial parts (foliage) of *M. longifolia* and *P. gnaphalodes,* and flowers of *A. wilhelmsii,* were collected respectively in Masabi, Sarayan (33° 51′ N, 58° 31′ E; 1500 m a.s.l.), Birjand suburbs (32° 50′ N, 59° 13′ E; 1500 m asl) and Sade, Ghaenat (33° 1′ N, 59° 14 ′ E; 1900 m asl), located in South Khorasan province, Iran, from May to July, 2009. The plant material was dried naturally on laboratory benches at room temperature (23– 27 °C) until crisp. The dried material was stored at -24 °C, and then hydrodistilled to extract its essential oil. Essential oil was extracted from the plant samples using a Clevenger-type apparatus where the plant material is subjected to hydrodistillation. Conditions of extraction were 50 g of samples, 1:10 plant material/water volume ratio, and a four-hour distillation. The oil was dehydrated with anhydrous sodium sulphate (10 min), and immediately stored in airtight glassware in a refrigerator at 4 °C.

### Gas chromatography-mass spectrometry

The essential oils were analyzed on a gas Chromatograph mass spectrometer (Shimadzu -17A-QP5050, Japan). The gas chromatography column was a super CP-SiI 5CB capillary column (50 m × 0.32 mm ID, 0.25 pm film thickness). The column oven temperature was set at 70 °C for 1 min, increased to 100 °C at a rate of 1.5 °C/min, increased to 180 °C at a rate of 4 °C/min, and held at 180 °C for 1 min. Next, it was increased to 200 °C at a rate of 10 °C/min, increased to 250 °C at a rate of 2.5 °C/min, and held at 250 °C for 5 min. Injector and detector temperatures were 280 °C and 300 °C respectively. The gas chromatography mass analysis was carried out with the same characteristics as used in gas chromatography. The ionization energy was 70 eV, with a scan time of 1 sec, and a mass range of 40–300 amu. Unknown essential oil was identified by comparing its gas chromatography retention time to that of known compounds, and its mass spectra to known compounds or published spectra.

### Fumigant toxicity

To determine the fumigant toxicity of the oil, glass vials (volume 70 mL) were prepared, each containing 10 adults (1–7 days old of undefined sex) of each species. Filter papers (2 cm diameter) were prepared by adding 5, 15, or 30 µl of oil to individual papers (without using any solvent). Then, each filter paper was attached to the under-surface of a screw cap and the cap was screwed tightly on the vial in order to generate concentrations of 71.43, 214.29, and 428.57 µl/L air, respectively. Each concentration and control was replicated five times. Mortality was determined 3, 6, 9, 12, and 24 hours after exposure. When no signs of leg or antennal movement were observed, insects were considered dead.

Another experiment was designed to assess insect mortality using 50% lethal doses (LC_50_). The concentrations of the essential oils were chosen based on range-finding tests (to cause mortality between 5 and 90%). For each bioassay, five different concentrations, each with five replicates and ten individuals per replicate, were used. The volumes of the glass vials were 300 mL and 500 mL for *T. castaneum* and *C. maculatus,* respectively. The dead and living insects in each bottle were counted 24 hours after initial exposure to the essential oil. The mortality was determined as described in the previous experiment. The treatment bottles were monitored for 48 hours after recording the data, and no affected insect recovered. Data obtained from each dose response bioassay were subjected to probit analysis. LC_50_ values were determined by log-probit regression using SPSS 16.0 for Windows.

## Results and Discussion

### Chemical composition of essential oils

The chemical composition of the essential oils of the studied plants, *M. longifolia* and *P. gnaphalodes* aerial parts, and *A. wilhelmsii* flowers, are presented in Table 1.

In *M. longifolia,* the major compounds were piperitenone (43.9%), tripal (14.3%), oxathiane (9.3%), piperitone oxide (5.9%), and d-limonen (4.3%). The major compounds of the Iranian *M. longifolia* oil were piperitone (43.9%), limonene (13.5%), and transpiperitol (12.9%) (Rasooli and Rezaei 2002). However, identification of piperitone as the major compound in the *M. longifolia* oil is in sharp contrast to other reports where the oil had carvone (Monfared et al. 2002) or ciscarveol (Zeinali et al. 2005) as the major component.

The analysis of *P. gnaphalodes* essential oil revealed that chrysanthenyl acetate (22.38%), 2L -4L-dihydroxy eicosane (18.5%), verbenol (16.59%), dehydroaromadendrene (12.54%), β-pinen (6.43%), and 1,8 cineol (5.6%) were the main products. Weyerstahl et al. (1999) reported the oil prepared from aerial parts of *P. gnaphalodes* collected in the Elbrus mountains, Tehran province, Iran contained about 65% monoterpenes, with α-pinene (34%) and 1,8-cineole (12%) as main compounds, and β-pinen (0.6%), alloaromadendreneand (0.4%) and trans-verbenol (0.2%) as minor compounds. Also, cischrysanthenol (oxidized monoterpenes) (2.3%) and its esters cis-chrysanthenyl formate (2.9%), cis-chrysanthenyl acetate (0.2%), chrysanthenone (monoterpene) (2%), and its related product isochrysanthenone (0.7%) were identified in the oil of this plant. Chrysanthenyl acetate was reported as the main component in the oil of *Artemisia vulgaris* L. collected from some localities in North Lithuania (Judžzentienè and Buzelytè 2006), *Tanacetum balsamita* subsp. *balsamita* (Asteraceae) from Turkey (Bagci et al. 2008b), and *Tanacetum parathenium* (L.) from England and the Netherlands (Hendriks et al. 1996; Christensen et al. 1999). However, there are no data in the literature on the prevalence of chrysanthenyl acetate, 2L-4Ldihydroxy eicosane, verbenol, and dehydroaromadendrene in *P. gnaphalodes* essential oil.

**Figure 1.**
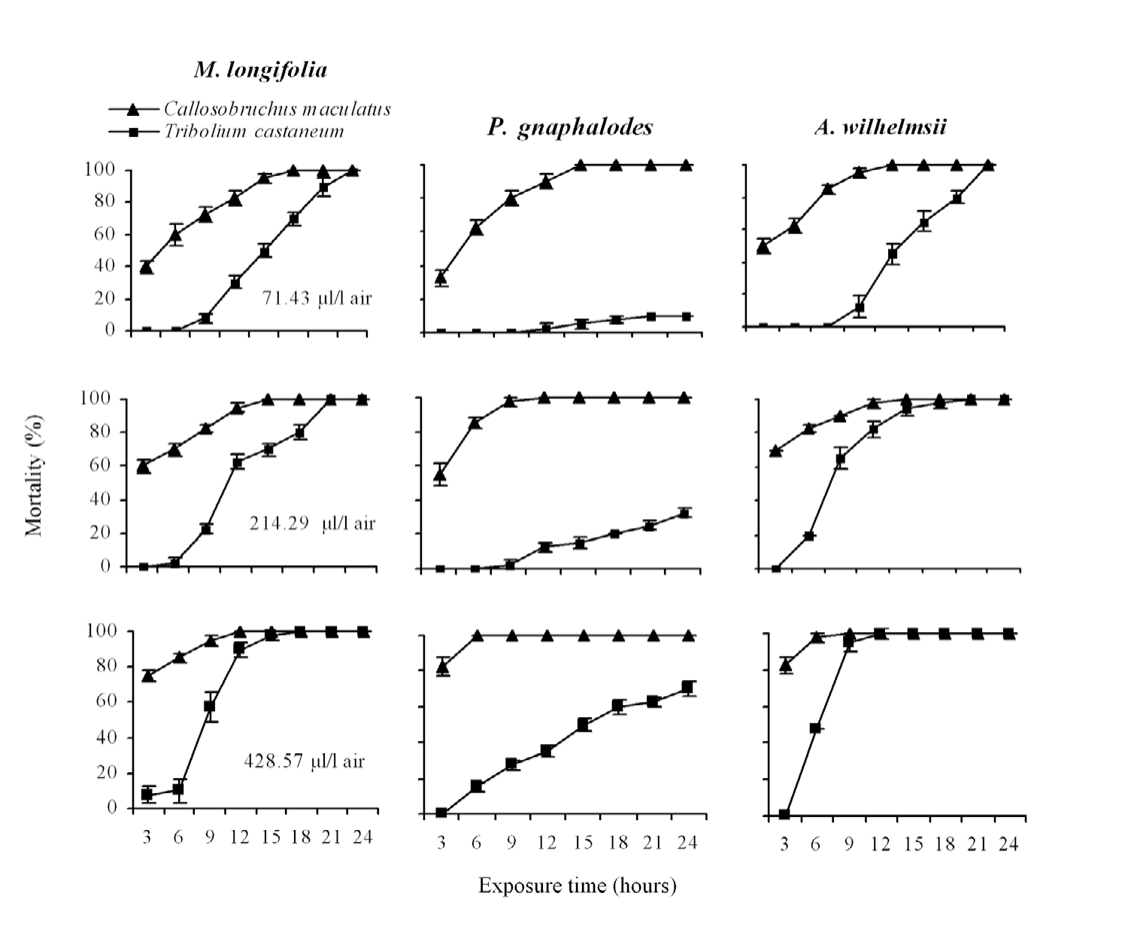
Mean (5 replications, 10 individuals each) cumulative percentage mortality of *Tribolium. castaneum* and *Callosobruchus maculatus* exposed to various concentrations of essential oils from *Mentha longifolia* and *Pulicaria gnaphalodes* aerial parts and *A. wilhelmsii* flowers at various periods of time. High quality figures are available online.

Gas chromatography-mass spectrometry analysis indicated that there are 20 major compounds in the oil of *A. wilhelmsii* flowers, comprising 88% of the total weight. 1,8-cineole was the most abundant compound (13.03%), followed by caranol (8.26%), α pinene (6%), farnesyl acetate (6%), p-cymene (6%), together with lesser amounts of the other important insecticidal compounds, including camphor (4.2%), carvacrol (3.7%), and terpineol (3.1%). The results of our analysis are in agreement with previous reports that have also reported carvacrol, 1,8-cineol, camphor, and α-pinene as the major components of the oil of *A. wilhelmsii* from Iran (Afsharypour et al. 1996; Javidnia et al. 2004; Ghani et al. 2008) and Turkey (Bagci et al. 2008a).

### Fumigant toxicity

In all cases, considerable differences in insect mortality due to essential oil vapor were observed using different concentrations and exposure times. The mortality increased with rising concentrations and exposure time (Figure 1). Results indicated that the oils of all three plants were significantly more toxic against *C. maculatus* than *T. castaneum,* as inferred by the confidence intervals of LC_50_ (Figure 1). Furthermore, a difference in the response of the insect species to the essential oils has previously been reported for storedproduct insects (Lee et al. 2003; Negahban et al. 2007).

Based on LC_50_ (Table 1) and fumigant toxicity experiments (Figure 1), the oils of the three plants displayed the same strong insecticidal activity against *C. maculatus* (between 1.54 µl/L air in *P. gnaphalodes,* and 2.65 µl/L air in *A. wilhelmsii).* While the oils of *A. wilhelmsii* and *M. longifolia* showed the same strong insecticidal activity against *T. castaneum* (LC_50_=10.02 and 13.05 µl/L air, respectively), the oil of *P. gnaphalodes* showed poor activity against the insect (LC_50_=297.9 µl/Lair) (Table 2).

No study has been previously reported on the insecticidal activities of the oils of three tested plants against *C. maculatus* and *T. castaneum;* however, it has been reported that the oils and extracts of the tested plants had insecticidal activity against other insects. For example, the ethanol extracts from the leaves of *M. longifolia* revealed insecticidal activity with a maximum of 70% mortality six days after grain pollution (5 grams of plant powder extracted with 500 mL ethanol, diluted to 10%, and used at an amount of 4 mL per 20 grams of grains in plastic vials with 100 mL capacity) (Saljoqi et al. 2006). Contact and fumigant insecticidal actions of *Achillea wilhelmsii* (Calmasur et al. 2006) and other *Achillea* species (Jovanovic et al. 2007; Magdy and Samir 2008) have been demonstrated against stored product pests. One hundred percent mortality was achieved with 2 µl/L air doses of the essential oils extracted from the leaves of *A. wilhelmsii* against *Sitophilus granarius* after an exposure time of 48 hours. However, mortality of *Tribolium confusum* at the same condition was about 90% (Calmasur et al. 2006).

Monoterpenes have been well documented as active fumigants, repellents, and insecticides toward stored-product insects (Papachristos et al. 2004). The insecticidal activity of the essential oils investigated in our study may be attributed to their having major monoterpenes components, because some major compounds of the test oils, such as carvacrol, camphor, 1,8-cineole, α-pinene, p-cymene, piperitenone oxide, and terpineol possessed insecticidal effects against the test insects (Traboulsi et al. 2002; Papachristos et al. 2004; Miresmailli et al. 2006; Cetin et al. 2007; Kordali et al. 2008; Magdy and Samir 2008).

The essential oils from these plants could become a viable alternative to conventional chemical control strategies. However, further studies need to be conducted in order to evaluate the safety of these oils before practical use in stored-product insect control.

**Table 1.**
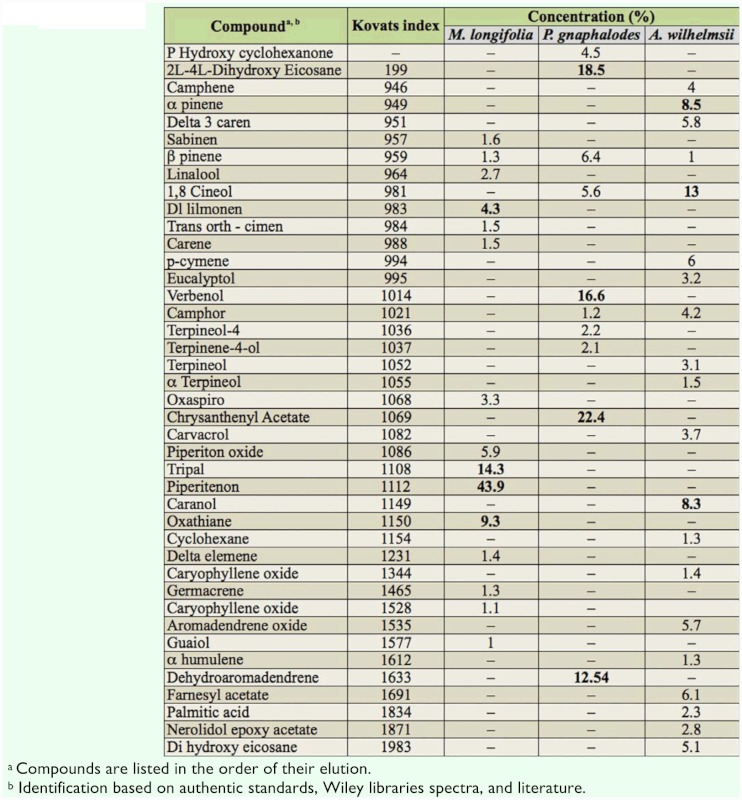
Chemical composition of essential oils of *Mentha longifolia* and *Pulicaria gnaphalodes* vegetative parts and *Achillea* *wilhelmsii* flowers.

**Table 2.**
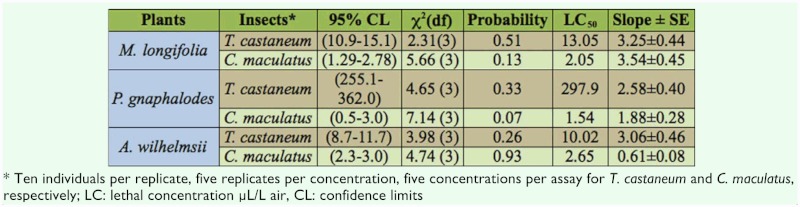
Efficiency of essential oil extracted from three plants against *Tribolium castaneum* and *Callosobruchus maculatus* adults.
